# Id1 and NF-κB promote the generation of CD133^+^ and BMI-1^+^ keratinocytes and the growth of xenograft tumors in mice

**DOI:** 10.3892/ijo.2014.2309

**Published:** 2014-02-21

**Authors:** JINHUO LAI, QIAN CAI, MERRILL A. BIEL, CHUAN WANG, XIAOHUA HU, SHAOYUAN WANG, JIZHEN LIN

**Affiliations:** 1 Department of Oncology of Union Hospital, Institute of Immunotherapy, Fujian Medical University, Fuzhou, P.R. China;; 2 Department of Otolaryngology, Head and Neck Surgery, University of Minnesota School of Medicine, Minneapolis, MN, USA;; 3 Department of Otolaryngology, Sun Yat-sen Memorial Hospital, Sun Yat-sen University, Guangdong, P.R. China;; 4 Ear, Nose and Throat Specialty Care of Minnesota, Minneapolis, MN, USA

**Keywords:** oral squamous cell carcinoma, head and neck cancer, CD133^+^ BMI-1^+^ keratinocytes, cancer stem-like cells, OCT4, Id1, NF-κB

## Abstract

Id1 and NF-κB are highly expressed in oral squamous cell carcinoma (OSCC). Whether they have a synergistic role in the carcinogenesis of OSCC is unclear. The current study was designed to demonstrate the synergy of both Id1 and NF-κB in the underlying disease mechanisms of OSCC using *in vitro* and *in vivo* animal models. Id1 and NF-κB strengthened the expression of both CD133 and BMI-1 in OSCC cell cultures. CD133^+^ and BMI-1^+^ keratinocytes from OSCC tissues and cell cultures initiated the growth of xenograft tumors in SCID/Beige mice. Id1 and NF-κB regulate the expression of CD133 and BMI-1 in an additive or synergistic manner in OSCC, which is associated with the generation of naïve and self-renewable keratinocytes and initiate the growth of xenograft tumors *in vivo*.

## Introduction

Emerging evidence indicates that oral squamous cell carcinoma (OSCC) harbors cancer initiating cells (1–6). However, it is unclear what subpopulations initiate the growth of OSCC in animals and clinical patients. Our recent studies indicate that head and neck cancer including OSCC is characterized by the expression of Id1 and NF-κB in the majority of clinical patients by microarrays (7,8) and cancer cells, rich in these two proteins are by nature aggressive and have poor clinical outcomes (9–12).

Id1 has been shown to trigger the xenograft tumor growth in nude mice via the regulation of the PI3K/Akt and NF-κB/Survivin pathways (8). NF-κB is also tumorigenic when combined with sv40 in keratinocytes (13) or Id1 (8). Approximately 60% of head and neck cancer specimens are positive for Id1 and NF-κB proteins. The *in vivo* data from our previous studies indicate that both Id1 and NF-κB are highly expressed in the edge of xenograft tumor lumps and are highly proliferative *in vitro* (8). Biologically, Id1 is involved in the immortalization of differentiated keratinoyctes (14), increases cellular proliferation when associated with NF-κB (15), yielding fast-growing keratinocyte spheres with naïve properties. Therefore, Id1 and NF-κB are important oncogenic proteins in keeping cells in a state of naiveness and proliferation and thought to contribute to the generation of cancer initiating cells.

Cancer initiating cells are side populations with the capability to initiate cancer growth in the body (16–18). These naïve side populations are capable of differentiating into a variety of cancer cell phenotypes and recapitulating the tumor heterogeneity in animal models (19–21). In general, naïve cancer cells are marked with stem cell markers such as CD133 and are more resistant to radiation and chemotherapy than other cancer cell populations (22,23).

CD133 is a glycoprotein, also known as Prominin 1 (PROM1), in humans and rodents (24,25). It is the founding member of pentaspan transmembrane glycoproteins, specifically associated with plasma membrane protrusions, irrespective of the cell type. CD133 is expressed in hematopoietic stem cells, neural stem cells, and some other cell types (26–30). CD133^+^ cells are thought to be glioma initiating cells in the brain, with its tumorigenicity much higher than than CD133^−^ cells (31,32). Interestingly, CD133+ cells are extensively present in the clinical head and neck squamous cell carcinoma specimens and possess stem cell-like properties *in vitro* (33) and promote the initiation of head and neck cancer (34).

BMI-1 is a key transcription factor that immortalizes human mucosal keratinoyctes (35), which is a hallmark of cancer cells (36). It is unclear whether BMI-1 is involved in the self renewal of OSCC. It has been observed that BMI-1 is expressed in clinical OSCC specimens but not in normal mucosal controls.

In this study, we hypothesized that CD133^+^ and BMI-1^+^ keratinocytes via Id1 and NF-κB subunit p65 in OSCC are capable of initiating xenograft tumors in immunodeficient mice. To verify this hypothesis, we isolated CD133^+^ and BMI-1^+^ keratinocytes from fresh OSCC tissue cultures and CA9-22 cell cultures and then injected them into SCID/Beige mice. It was found that CD133^+^ and BMI-1^+^ cells, either from OSCC tissues or CA9-22 cell cultures, initiated the xenograft tumor growth in SCID/Beige mice whereas CD133^−^ cells did not. The xenograft tumors from these CD133^+^ and BMI-1^+^ keratinocytes possessed similar phenotypes as those of the OSCC clinical samples.

## Materials and methods

### Clinical specimens

The surgical specimens were collected from clinical patients who underwent surgery at the Union Hospital, Fujian Medical University, Fuzhou; Sun Yatsen Memorial Hospital, University of Sun Yatsen, Guangzhou, China; and the Department of Otolaryngology, University of Minnesota Hospital and Clinics, Minneapolis, MN, USA. The expression of mRNA transcripts was interrogated with the database of microarrays in terms of the genes of interest in the present study. Control specimens were biopsies of normal tissues close to the cancer site. Seven OSCC surgical specimens and five normal tissues were used for western blot analysis. An additional five OSCC fresh tissues were xenograft tumor passages in SCID/Beige mice first and then harvested for isolation of CD133^+^ and BMI-1^+^ cells and subsequent cell cultures. All specimens and clinical data in this study were procured, handled and maintained according to the protocols approved by each Institutional Review Board (IRB), e.g., the University of Minnesota, the Fujian Medical University and the Sun Yatsen University.

### Cell cultures

Surgical OSCC tissues were obtained from clinical patients at the University of Minnesota Hospitals and Clinics (UMHC), Sun Yatsen University Hospital and the Union Hospital of Fujian Medical University. Fresh tissues were first implanted into the flank of mice to allow for tumor growth and then passed into SCID/Beige mice generation after generation. Xenograft tumors were dissected out and immersed into tissue culture media. Tissue cells were dissociated by using a cell isolator (Gentle MACS, Miltenyi Biotec, Auburn, CA, USA). CD133^+^ cells were then isolated by MACS with CD133 monoclonal antibody beads cultured in RPMI-1640 (Life Technologies, Invitrogen, Carlsbad, CA, USA) on glass chamber slides and regular cell culture dishes. The expression of BMI-1 was determined by reverse-transcription-polymerase chain reaction (RT-PCR), western blot analysis, and fluorescence activated cell sorting (FACS) from these CD133^+^ cell cultures.

### Construction of keratinocytes with stable expression of both ID1 and NF-κB

HOK16B is a cell line derived from keratinocytes in the oral cavity immortalized with human papillomavirus (37); CA9-22, a cell line established from an oral cancer patient was maintained in RPMI-1640 (Life Technologies, Invitrogen), HOK-16B was maintained in keratinocyte basal medium (Lon2a) and the CD133 cDNA was prepared as previously described (31). Efficacy of Id1 cDNA expression after transient transfection was determined by inverted microscopy or flow cytometry.

### Reverse transcription-polymerase chain reaction (RT-PCR)

Total RNA was isolated from five clinical OSCC tissues and controls using the RNA^®^ Miniprep kit (Stratagene, Santa Clara, CA, USA). Residual genomic DNA in total RNA samples was digested with DNases according to the manufacturer’s instruction. Specific primers for the human CD133 (5′-ctactatgaagc agggatta-3′/5′-aacgcctctttggtctcctt-3′) and BMI-1 (5′-cttggctcgc attcattttc-3′/5′-tcacctcctccttagatttc-3′) were used for this study, using the method as described previously (26).

### Immunohistochemistry

Cell cultures from clinical OSCC specimens were performed as previously described. Cells were fixed in 100% alcohol and incubated for 90 min with CD133 (AC133, Miltenyi Biotec) and then incubated with secondary antibodies, FITC- or TRITC-conjugated mouse anti-rabbit, goat anti-mouse IgG (Zymed, San Francisco, CA, USA), as previously described (8). Later on, cellular nuclei in the same slide were stained with 4′,6′-diamidino-2-phenylindole (DAPI) after the antibody stain became faint. This allows cellular nuclei, CD133 and BMI-1 proteins to be shown simultaneously by using Photoshop software to evaluate the co-expression of both CD133 and BMI-1 in cultured OSCC cells.

### Western blot analysis

CD133 and BMI-1 proteins in OSCC tissues were detected with an Enhanced Chemiluminescence Western Blotting Detection Kit (Amersham Pharmacia Biotech, Piscataway, NJ, USA) after binding of mouse anti-human CD133/1 (AC133) MAb (Miltenyi Biotec) and goat anti-human BMI-1 (sc30943, Santa Cruz Biotechnology, Santa Cruz, CA, USA). Horseradish peroxidase (HPR)-conjugated anti-mouse and anti-goat secondary IgG antibodies were used (Santa Cruz Biotechnology). Anti-CD44 antibody (ab41478, Abcam, Cambridge, MA, USA), anti-CD1 (ab95587, Abcam), anti-Cdc2 (1:1,000 dilution, 9112S, Cell Signaling Technology, Danvers, MA, USA), and anti-GAPDH antibody (ab9485, Novus Biologicals, Littleton, CO, USA) were used for western blot analysis. Cycloheximide at a concentration of 10 *μ*M was used for inhibition of protein synthesis in CA9-22 cell cultures. Catharidic acid (CA, CAS#28874-45-5, 1 *μ*M) and arginine vasopressin (AVP, CAS#113-79-1, 10-6 mol) were used for inhibition of the Cdc2 protein and AVP protein, respectively, after cells were transfected with Id1+p65. CH, CA and AVP were all purchased from Sigma-Aldrich (St. Louis, MO, USA).

### Fluorescence activated cell sorting (FACS) analysis

Briefly, cells were washed with phosphate-buffered saline (PBS), harvested by trypsinization, and incubated with PE-conjugated anti-human CD133 (130-080-901, Miltenyi Biotec), APC-conjugated anti-human CD44 (559942, BD Biosciences Pharmingen, San Diego, CA, USA), and FITC-conjugated anti-human CD24 (555427, BD Biosciences Pharmingen) antibodies. For BMI-1 analysis, cells were washed with PBS, harvested by trypsinization, pre-incubated with 0.3% saponin in PBS for 10 min, and then incubated with goat anti-human BMI-1 (CAT#sc-30934, Santa Cruz Biotechnology), followed by secondary mouse anti-goat antibody (FITC conjugated). In a similar way, cells were stained with anti-Id1 (sc-488, Santa Cruz Biotechnology) and anti-BMI-1 (sc-30943, Santa Cruz Biotechnology) antibodies. Cells were resuspended in PBS and analyzed on FACSCalibur using CellQuest Pro (BD Biosciences) and FlowJo 7.6 (Tree Star, Inc., Ashland, OR, USA). Similarly, Id1^+^ and BrdU^+^ cells were analyzed with flow cytometry as above.

### Cell sorting

The dissociated cells were incubated with PE-conjugated anti-human CD133 at 20 *μ*l per 10^6^ cells for 20 min, at 4˚C, in the dark and washed three times with excessive PBS. In a final labelling step, 80 *μ*l MACS buffer (PBS supplemented with 0.5% BSA and 2 mM EDTA) per 10^7^ cells and 20 *μ*l anti-PE MACS Microbeads^®^ (Miltenyi Biotec) per 10^7^ cells were added to the cell pellet. After mixing, the cells were incubated in the dark for 15 min at 4˚C. The cells were washed with excess MACS buffer and resuspended in 500 *μ*l buffer per 10^7^ cells. The magnetic separation of the cells was performed using an LS-column (Miltenyi Biotec) that was placed in a VarioMACS separator (Miltenyi Biotec), as described by the manufacturer’s protocol. The positive selected cells were finally suspended in 1 ml of MACS buffer per 4×10^6^ total cells. Id1^+^ cell subpopulation was sorted out by using the same protocol as above. Id1 antibody was conjugated with FITC.

### Trypan blue exclusion

Briefly, cells were transfected in transfection medium with Id1+p65, p65, Id1, and empty vector at 1.4 *μ*g/ml for 16 h, recovered in cell culture medium for 24 h, and then harvested for evaluation of cell numbers after staining with a (trypan blue) dye. Cells were washed first in PBS and incubated in 0.3 ml of 0.05% trypsin-EDTA solution for 10 min. Trypsin solution (5 *μ*l) was mixed with 5 *μ*l of trypan blue and transferred to a hemacytometer for cell counting. Results are presented as 10^4^ viable cells.

### Cell cycle progression

For cell cycle progression analysis, cells were cultured in 6-well plates till 60% confluence, transfected with empty vector, Id1, p65 and ID1+p65 at 1.4 *μ*g/ml for 16 h, and then recovered in full culture media for 24 h, washed with PBS, dissociated with trypsin-EDTA, washed with ice cold PBS, fixed with 100% ethanol, and stored at −20˚C until analysis. Approximately 5×10^4^ cells were resuspended in 100 *μ*l of 40 *μ*g/ml DNase-free RNase A with 100 *μ*l of propidium iodide (stock solution 200 *μ*g/ml) and incubated at room temperature for 30 min. The fluorescence excited by FL-2 was measured on a FACSCalibur (BD Biosciences). The cell cycle progression of approximately 10^4^ CA9-22 cells was analyzed on CellQuest Pro (software version 4.0 Becton-Dickinson, Franklin Lakes, NJ, USA). The experiment was performed in triplicate. Data are presented as means ± SD (n=3).

### Xenograft tumor growth in SCID/Beige mice

SCID/Beige mice were used in this study. CD133^+^ and BMI-1^+^ keratinocytes from both fresh OSCC tissue cultures and CA9-22 cell cultures were, respectively, injected into 8 SCID/Beige mice in the flank back skin subcutaneously with 100, 1,000, 10,000, and 100,000 viable cells per injection site, namely, groups I, II, III and IV with the above cell numbers being injected. After injection, tumor volume in SCID/Beige mice was measured routinely by width, length and height of xenograft tumors in a three-dimension manner on a biweekly basis up to two months. Tumor weight was measured when animals were sacrificed for harvesting xenograft tissues.

### Statistical analysis

The Student’s t-test was used for evaluation of differences between controls and experiments *in vitro*. P-values <0.05 were considered to indicate a statistically significant difference.

## Results

### CD133 and BMI-1 proteins are co-expressed in OSCC tissue cultures

**Figure 1.**
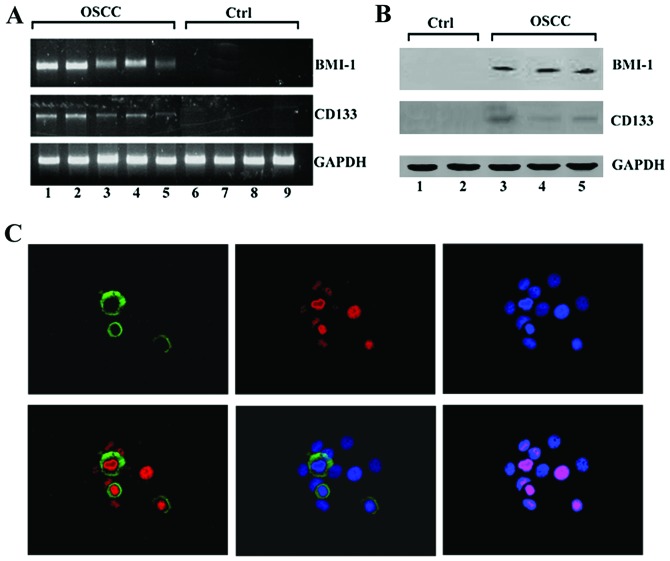
The expression of CD133 mRNA transcripts and proteins in OSCC specimens and cell cultures. (A) The CD133 and BMI-1 mRNA transcripts were detected in the surgical OSCC specimens (lanes 1–5) but not in the control tissues (lanes 6–9). (B) The CD133 and BMI-1 proteins were found in the clinical OSCC specimens but not in the control tissues. (C) Co-expression of both CD133 and BMI-1 proteins in cultured OSCC cells. Note that few cells are positive for CD133 (on cell surface, red) and BMI-1 in cell nuclei. Cell nuclei were stained blue with DAPI.

RT-PCR demonstrated the expression of both CD133 and BMI-1 mRNA transcripts in OSCC clinical specimens (Fig. 1A). Western blot analysis verified the expression of BMI-1 in the surgical specimens of OSCC (Fig. 1B). Immunohistochemistry demonstrated the co-expression of the both CD133 and BMI-1 proteins in cultured cells from clinical OSCC tissues (Fig. 1C).

### CD133 and BMI-1 increase in CA9-22 cell cultures transfected with Id1 and NF-κB

**Figure 2.**
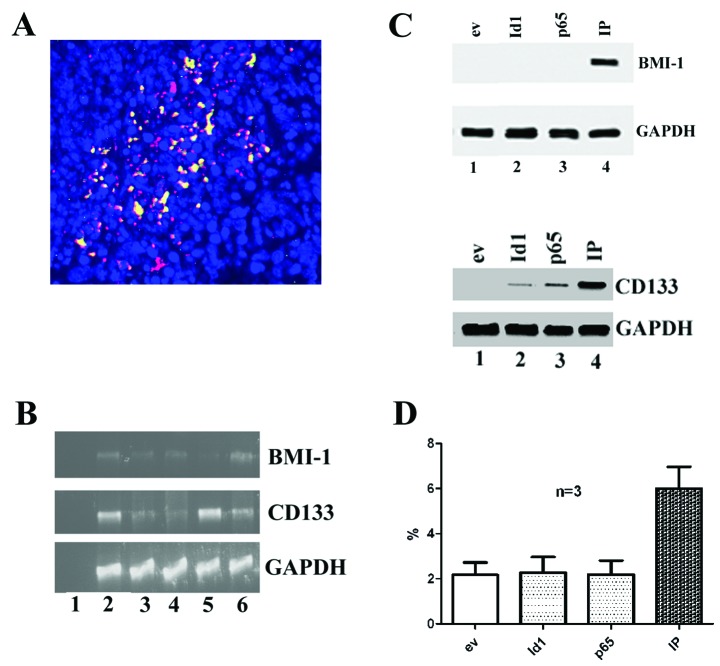
The regulation of both BMI-1 and CD133 in OSCC cells. (A) The Id1 and NF-κB subunit p65 were expressed in the clinical OSCC tissue sections. (B) RT-PCR demonstrated that both CD133 and BMI-1 mRNA transcripts were faintly detected in OSCC tissue cultures. (C) In cultured CA9-22 cells, BMI-1 was undetected in controls but detected in both Id1 and p65 transfected cells (3 days after transfection). CD133 was not detected in controls but detected in Id1 and p65 transfected cells, obsviously upregulated by both Id1+p65 (IP). (D) IP synergistically increased the percentage of BMI-1^+^ cells, but not Id1 and p65 alone by FACS in OSCC tissue cultures. ^*^p<0.05.

Id1 and NF-κB subunit p65 were detected in OSCC specimens (Fig. 2A). It is noted that some OSCC cells express both Id1 and NF-κB subunit p65 (yellow color indicating the co-expression of both Id1 and p65, red indicating Id1 positive cells and green p65 positive cells). The CD133 and BMI-1 mRNA transcripts were detected in OSCC tissue cultures by RT-PCR (Fig. 2B). In the cell cultures of CA9-22, the expression of BMI1 protein was not detected in Id1 or p65 transfected cells alone but detected in cells with the transfection of both Id1 and p65 in combination. While the expression of CD133 protein was not detected in empty vector transfected cells, but it was detected in cells with the transfection of Id1, p65 or Id1+p65 (Fig. 2C). The BMI-1 protein amount increased and became detected when cells were transfected with both Id1+p65, but were not detected with Id1 and p65 alone in OSCC tissue cultures (Fig. 2D).

### Id1 and BMI-1 are co-expressed in proliferating OSCC cells

**Figure 3.**
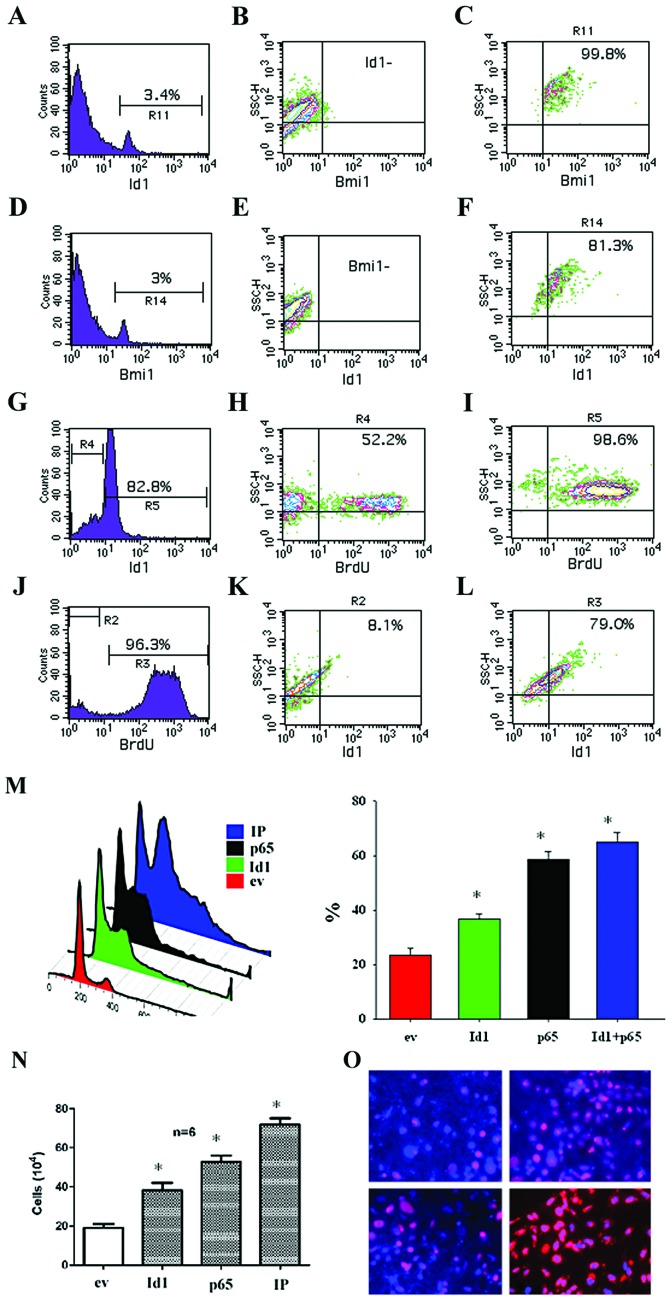
The relationship between Id1^+^ and BMI-1^+^ cells as well as Id1^+^ and BrdU+ in OSCC cells. Cells were stained with both Id1 and BMI-1 antibodies and examined by FACS. (A) In OSCC cells, as indicated by marker R11, there were 3.4% of Id1^+^ cells. (B) Approximately 97.6% of total cells were Id1^−^ and these cells were also BMI-1^−^. (C) Among Id1^+^ cells, 99.8% were BMI-1^+^. (D) In a similar way, as measured by marker R14, there were approximately 3% of BMI-1^+^ cells. (E) Among BMI-1^−^ cells, accounting for 97% of total cells, were also Id1^−^. (F) Among the Id1^+^ cells, they were 81.3% BMI-1^+^. In the Id1^+^ subpopulation, there were 82.8% of cells remaining Id1^+^ after culture for 24 h (G). In the Id^−^ cells, 52.2% of cells remained BrdU^+^ (H) whereas in the Id1^+^ cells 98.6% of cells remained BrdU^+^ (I). Accordingly, in the Id1^+^ cell cultures, there were 96.3% of BrdU^+^ cells (J). In the BrdU^−^ cells 8.1% of cells were Id1^+^ (K) whereas in the BrdU^+^ cells 79.0% of cells were Id1^+^ (L). Transfection with ev, Id1, p65 and IP indicated that Id1, p65, and IP significantly increased the cell cycle progression (M), cell counts (N), and BrdU incorporation (O) of CA9-22 cells. ^*^p<0.05.

Id1 is known to antagonize the differentiation of cells and make cells naïve whereas BMI-1 is involved in the self-renewal of keratinocytes. To study whether Id1 and BMI-1 are actually expressed in the same cell subpopulation, we performed FACS analyses after staining cells with both Id1 and BMI-1 specific antibodies. It was found that 3.4% of total cells were Id1^+^ and 96.6% of total cells were Id1^−^ (Fig. 3A). Among the Id1^−^ cells, there were almost 99.9% BMI-1^−^ (Fig. 3B). While among the Id1^+^ cells, there were approximately 99.8% BMI-1^+^ (Fig. 3C). In a similar way, approximately 3% of total cells were BMI-1^+^ (Fig. 3D). Among the BMI-1^−^ cells, there were nearly 100% Id1^−^ (Fig. 3E). While among the BMI-1^+^ cells, there were 81.3% of Id1^+^ cells (Fig. 3F). To study whether Id1 is involved in cell proliferation, we examined BrdU incorporation on the Id1^+^ subpopulation. It was found that of these Id1^+^ cells following cell cultivation for 24 h, 82.8% of cells remained Id1^+^ and 18.2% of cells became Id1^−^ (Fig. 3G). Among the Id1^−^ cells, there were 52.2% of BrdU^+^ cells (Fig. 3H). Among these Id1^+^ cells, 98.6% of cells were BrdU^+^ (Fig. 3I). In the Id1^+^ cells following cell cultivation for 24 h, there were 3.7% of cells remaining BrdU^−^ and 96.3% of cells were BrdU^+^ (Fig. 3J). Among the BrdU^−^ cells, there were 8.1% of BrdU^+^ cells (Fig. 3K). Among these BrdU^+^ cells, there were 79.0% of Id1^+^ cells (Fig. 3L). The effect of both Id1 and p65 on cell cycle progression and cell counts of CA9-22 cells was studied. It was found that Id1 and p65 increased the cell cycle progression (Fig. 3M), cell counts (Fig. 3N), and BrdU incorporation (Fig. 3O) of CA9-22 cell cultures. FACS data verified that both Id1 and BMI-1 were co-expressed in OSCC cells and Id1 increases BrdU^+^ cells.

### Id1 and p65 synergistically induce the expression of Bmi-1 in association with cyclin D1 and Cdc2 proteins

**Figure 4.**
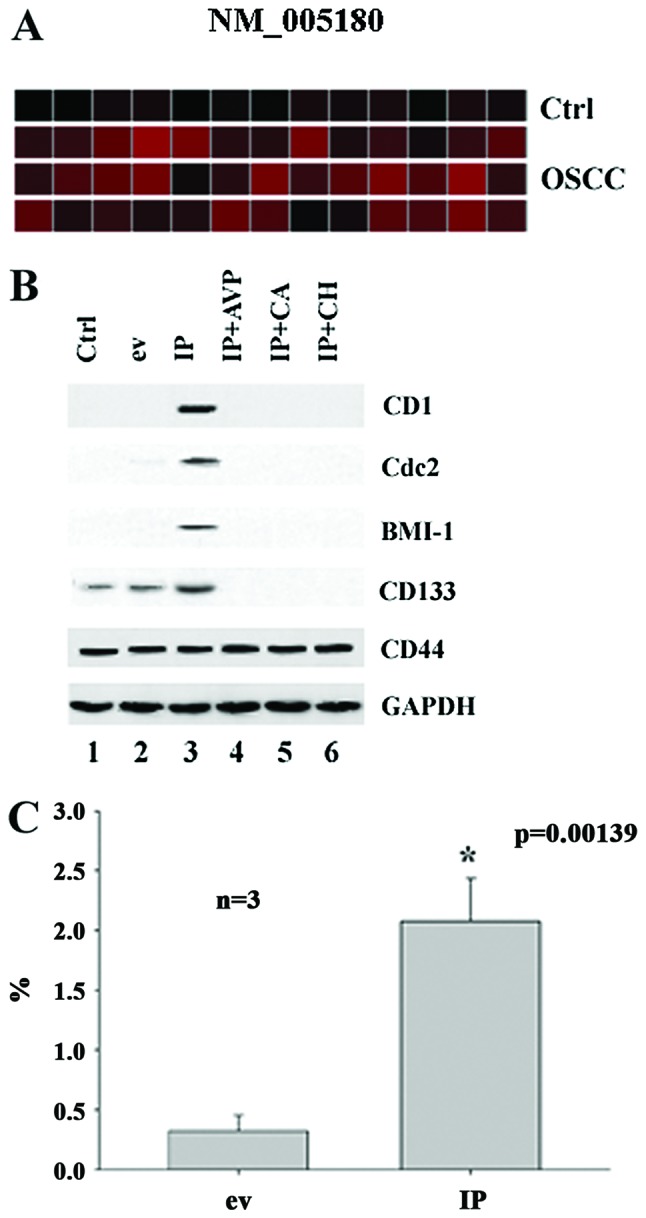
A–C, IP regulates the expression of BMI-1, and CD133 proteins possibly via the cyclin D1 and Cdc2 pathways in CA9-22 cells. IP-induced CD44 was not affected by incubating cells with AVP (cyclin D1 inhibitor) and catharidic acid (Cdc2 inhibitor) in CA9-22 cell cultures. While the expression of CD133, Bmi-1, Cdc2 and CD1 proteins were fully blocked by the addition of AVP, catharidic acid and cycloheximide (CH). This suggests that IP regulates the expression of Bmi-1, CD133 and CD44 via the cyclin D1 and Cdc2 signaling pathways. ^*^p<0.05.

As shown in Fig. 4A, BMI-1 was detected in the majority of the 39 OSCC specimens but not in 13 OSCC control tissues. To study whether Id1 and p65 are involved in the regulation of BMI-1, CD1 and Cdc2, we studied the expression of BMI-1, CD1 and Cdc2 in CA9-22 cells using molecular biological techniques. It was found that Id1+p65 synergistically induced the expression of Bmi-1, CD1, Cdc2 in CA9-22 cells (Fig. 4B). For verification of this finding, we transfected the cells with Id1 and p65 and examined the expression of Bmi-1. It was found that Id1+p65 significantly upregulated the expression of BMI-1 in CA9-22 cells by FACS (Fig. 4C). This indicates that Id1 and p65 synergistically regulate the expression of BMI-1 probably in association with the protein of CD1 and Cdc2 in OSCC cell cultures (CA9-22).

### CD133^+^ and BMI-1^+^ cells initiate xenograft tumors in SCID mice

**Figure 5.**
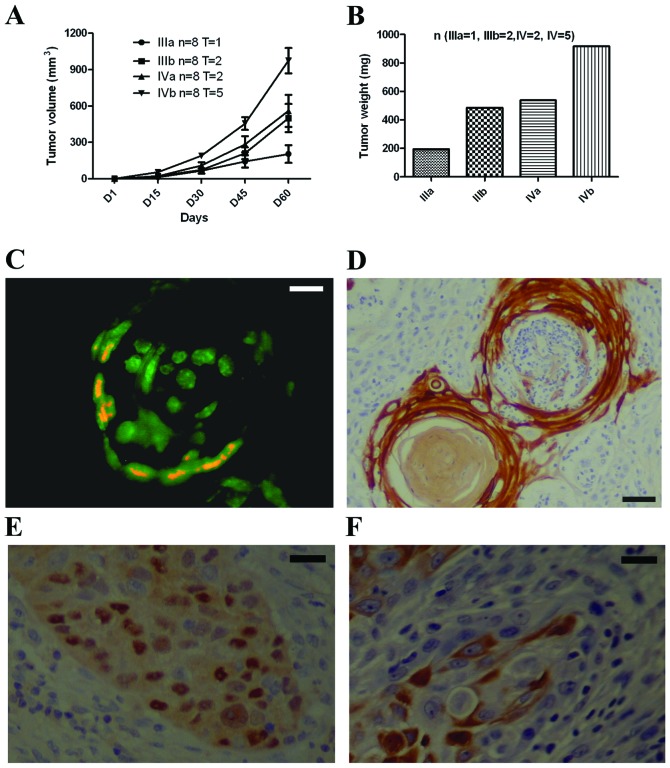
CD133^+^ and BMI-1^+^ cells initiate the growth of xenograft tumors in SCID/Beige mice. (A) Xenograft tumor volumes (mm^3^) increased with time. (B) At the end of the two-month experiment, a total of tumor weight was measured in the groups indicated. (C) Id1 and NF-κB subunit p65 were co-expressed in the edge of xenograft tumor tissue section (yellow in color pointing to the co-expression of both Id1 and NF-κB). (D) NF-κB subunit p65 was highly positive in the edge of xenograft tumors. (E) BMI-1 was positive in the nuclei of xengoraft tumors. (F) CD133 was positive in xenograft tissue sections. Bars in C, E and F, 10 *μ*m; bar in D, 20 *μ*m.

To study the tumorigenic potential of both CD133^+^ and BMI-1^+^ cells, we sorted out CD133^+^ cells from OSCC and CA9-22 cell cultures and then injected into SCID/Beige mice. These CD133^+^ cells are also BMI-1^+^ by FACS. At 100 and 1,000 cells, namely, groups I and II, there were no xenograft tumors in animals. At 10,000 CD133^+^ and BMI-1^+^ keratinocytes of CA9-22 and OSCC cells, there were one and two xenograft tumors, respectively, from CA9-22- and OSCC-injected animals. At 100,000 CD133^+^ and BMI-1^+^ keratinoyctes, there were two and five xenograft tumors, respectively, from CA9-22- and OSCC-injected animals (Fig. 5A and B). No xenograft tumors were found in animals with injection of CD133^−^ and BMI-1^−^ cells. Tissue sections were then stained with antibodies against Id1, NF-κB, CD133 and BMI-1. It was found that Id1 and NF-κB were co-expressed in the edge of xenograft tumors (Fig. 5C and D). It is noted that Id1 is expressed in the entire xenograft tumor lump whereas NF-κB is in the edge of xenograft tumors. BMI-1 was found in the nuclei of xenograft tumors (Fig. 5E) whereas CD133 was found in the membrane of xenograft tumors (Fig. 5F).

## Discussion

CD133^+^ and BMI-1^+^ keratinocytes exist in OSCC clinical specimens, tissue cultures, and OSCC cell lines such as CA9-22 as shown in this study. The CD133 positive cells are few so that the protein is not abundant (faint bands) by western blot analysis but clearly observed in tissue cultures with dual immunohistochemistry staining. CD133^+^ BMI-1^+^ keratinoyctes from both OSCC tissue cultures and CA9-22 cell cultures are capable of growing xenograft tumors in SCID/Beige mice. This confirms the importance of CD133^+^ and BMI-1^+^ subpopulation in the carcinogenesis of OSCC. It is becoming clear that there is a stem cell like subpopulation, that is, the CD133^+^ and BMI-1^+^ cells in OSCC specimens, OSCC tissue cultures, and OSCC cell lines, which is responsible for the initiation of OSCC.

This subpopulation is relevant to both Id1 and NF-κB subunit p65 which are frequently expressed in head and neck cancer including OSCC (8). In this study, we particularly examined the co-expression of both Id1 and p65 and it was found that both proteins are expressed in the OSCC tissue specimens (Fig. 2A). The *in vitro* studies indicate the synergy of both the Id1 and p65 proteins in OSCC to regulate the expression of BMI-1 (Fig. 2D). Id1 and p65 alone upregulated the expression of CD133 but was not sufficient for the upregulation of BMI-1. In the literature, the expression of the BMI-1 gene is related to the generation of naïve keratinocytes (38) and is also key to generation of stem cells in normal tissues (38–40). In OSCC, BMI-1 induction requires the synergy of both the Id1 and NF-κB subunit p65. In addition, the expression of BMI-1 is related to human papillomavirus (HPV) infection of the upper respiratory tract (35,41). HPV E6/E7 oncogenes correlate with the expression of Id1 (42). It appears that both Id1 and NF-κB are related to chronic inflammation in the upper respiratory tract.

The importance of this subpopulation lies in its potential to initiate the growth of xenograft tumors in SCID/Beige mice. In comparison with other subpopulations such as CD44^+^ subpopulation, CD133^+^ cells are indeed a minority cell subpopulation, accounting for less than 3% in total cell population, and more aggressive than others. With 1,000 cells, CD133^+^ and BMI-1^+^ subpopulations fail to grow any xenograft tumors in SCID/Beige mice whereas CD133^+^ and BMI-1^+^ cells at 100,000 succeeded in growing xenograft tumors in 62.5% of SCID/Beige mice. CD133^+^ and BMI-1^+^ keratinoyctes from the OSCC tissue cultures and CA9-22 cell cultures are linked with Id1 and p65 as well as cell proliferation and renewal.

Carcinogenesis of OSCC involves multiple signaling pathways. One is the Id1-CD133 pathway in which Id1 plays an important role in the naïve behavior of OSCC. In our previous study (8), we demonstrated that Id1 is involved in the upregulation of the PI3K/Akt and survivin pathways which promote the survival of cancer cells. In addition to cell survival, we now learn that Id1 also makes cancer cells naïve by increasing the expression of CD133. Apparently, CD133 is associated with BMI-1. These cell markers indicate their stemness of the CD133^+^ and BMI-1^+^ subpopulation when it is linked to the above signaling pathways (Id1 and NF-κB pathways) which are relevant to chronic inflammation.

Furthermore, the synergy of both Id1 and p65 in immortalized mucosal keratinocytes (HOK16B) resulted in the upregulation of BMI-1. This signaling pathway plays an important role in the self-renewal of immortalized mucosal keratinocytes. This may promote cells to a malignant level of keratinocytes, associated with chronic infections such as HPV in the larynx. Similar situation is also observed in nasopharyngeal carcinoma, as shown in our recent study, Id1 and NF-κB subunit p65 in nasopharyngeal cancer are important factors for prediction of clinically poor outcomes (9).

Recently, CD133 has been shown to be present in stem-like cells in head and neck squemous cell carcinoma (34). In addition, we have observed in our laboratory that Id1 regulates the expression of CD44 (data not shown). CD44 in head and neck cancer has been shown to be present in cancer stem-like cells in head and neck cancer (1). These data collectively suggest that Id1 is an important transcription factor for the carcinogenesis of OSCC by blocking the differentiation of mucosal keratinocytes and promoting the generation of naïve keratinocytes. NF-κB also contributes to this carcinogenesis in conjunction with Id1. Id1 usually potentiates the activity of NF-κB in keratinocytes (15,43). We have shown in our previous study that the Id1 and NF-κB genes are upregulated in approximately 60–70% of head and neck cancer specimens by microarrays and immunohistochemistry analyses (8).

In this study, we found that the CD133^+^ and BMI-1^+^ cells are upregulated in OSCC tissues, and act like cancer stem cells. These cells initiate the growth of xenograft tumors derived from OSCC specimens and CA9-22 cell cultures as well as HOK16B when transfected with Id1 and p65. Since the potential of this subpopulation is not as high as genuine cancer stem cells, it is called cancer stem-like cells. The capability to initiate the growth of xenograft tumors in SCID/Beige mice is certain, approximately 10,000 cells are required to grow xenograft tumors in SCID/Beige mice. We note that there are 1–3% of total cells in OSCC cell cultures positive for CD133 and BMI-1. Consistent with this study, CD133^+^ cells are estimated to be approximately 3-5% of the total cancer cell population in the clinical head and neck cancer specimens and several head and neck cancer cell lines of a referenced study (33).
